# Supplementary material for: Photo-triggered solvent-free metamorphosis of polymeric materials
Source: Nat Commun. 2017 Sep 11;8:502. doi: 10.1038/s41467-017-00679-1 (PMC5593861; doi:10.1038/s41467-017-00679-1)
Supplement: Supplementary file 1 — Supplementary Information [file 41467_2017_679_MOESM1_ESM.pdf]

Description of Supplementary Files

File Name: Supplementary Information
Description: Supplementary Figures, Supplementary Tables, Supplementary Methods and Supplementary References

File Name: Supplementary Movie 1
Description: Repeatable MAT experiment for network-PBAHABI. UV (365 nm) was directly irradiated to network-PBAHABI on the slide glass heated at 50 °C with hot plate.

File Name: Supplementary Movie 2
Description: Repeatable MAT experiment for network-PDMSHABI. The sample on the inverted vial was irradiated with UV (365 nm) at room temperature (25 °C).

File Name: Supplementary Movie 3
Description: Photo-triggered metamorphosis of network-PDMSHABI. The sample was deposited on the templates and UV (365 nm) was irradiated at room temperature (25 °C).

File Name: Peer Review File

Supplementary Methods

Materials

n-Butyl actylate (BA) was passed through a plug of alumina prior to use. 4-(Propargyloxy)benzaldehyde,[1] and 4-(allyloxy)benzaldehyde[2] were synthesized according to the procedures reported in the literatures. Benzil (99%, TCI), ammonium acetate (97%, TCI), 4,4'-dinonyl-2,2'-dipyridyl (dNbpy) (98%, TCI), sodium azide (99%, TCI), *N,N,N',N'',N''*-Pentamethyldiethylenetriamine (PMDETA) (99%, TCI), potassium ferricyanide (98%, Wako), pentaerytyritol (98%, TCI), hexamethylcyclotrisiloxane (D3) (98%, Aldrich), 1,5,7-Triazabicyclo[4.4.0]dec-5-ene (TBD) (98%, Aldrich), chlorodimethylsilane (98%, Aldrich), platinum(0)-1,3-divinyltetramethyldisiloxane complex solution (Karstedt's catalyst) (Pt~2% in xylene, Aldrich), and other reagents were used as received.

Synthesis of pentaerythritol tetrakis(2-bromoisobutyrate) (1).

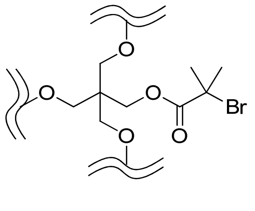

1

Into a flask was prepared pentaerythritol (7.4 g, 0.054 mol), TEA (61 mL, 0.435 mol), and CH_2Cl_2 (200 mL), and CH_2Cl_2 (100 mL) solution containing α-bromoisobutyryl bromide (100 g, 0.435 mmol) was slowly added at 0 °C. The mixture was stirred at room temperature for 23 h and sequentially washed with 5% aqueous NaOH, 1% aqueous HCl, and water. The CH_2Cl_2 phase was dried over $MgSO_4$, concentrated to dryness. The crude solid product

obtained was purified by recrystallization from ether. The yield was 33.6 g (85%). ^{1}H NMR (500 MHz, $CDCl_3$) δ ppm 1.94 (s, –C*H*$_3$), 4.33 (s, –C*H*$_2$–).

Synthesis of model small molecule having four lophines (2).

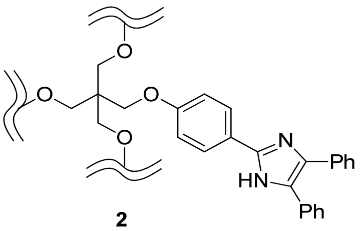

2

Into a flask, pentaerythrityl tetrabromide (2.0 g, 5.2 mmol), K_2CO_3 (17.0 g, 123 mmol), and 4-hydroxy benzaldehyde (5.0 g, 41 mmol, and DMF (25 mL) were added and the mixture was stirred at 100 °C for 90 h. After evaporating the solvent, the residue was diluted with $CHCl_3$ and poured into an excess amount of water. The organic was washed with water, dried over $MgSO_4$, and concentrated to dryness to give 4-(2,2-bis[(4-formylphenoxy)methyl]-3-(4-formylphenoxy)propoxy) benzaldehyde (**2**) as a white solid. The yield was 2.83 g (98%). ^{1}H NMR (500 MHz, $CDCl_3$) δ ppm 4.48 (s, 8H, –CH_2–), 7.05 (d, 8H, Ar*H*), 7.84 (d, 8H, Ar*H*), 9.89 (s, 4H, –C*H*O). A weight amount of 2 (1.0 g, 1.8 mmol), benzyl (1.90 g, 9.05 mmol), ammonium acetate (2.1 g, 27 mmol) and MeOH (20 mL) was added into a flask and the mixture was stirred at 60 °C for 18 h. After cooling to room temperature, a solid crystallized was collected by filtration washing with MeOH. The filtrate was concentrated and the crystallization procedure was repeated twice. The solid obtained was combined and dried under reduced pressure to afford 2,2'-(((2,2-bis((4-(4,5-diphenyl-1H-imidazol-2-yl)phenoxy)methyl)propane-1,3-

diyl)bis(oxy))bis(4,1-phenylene))bis(4,5-diphenyl-1H-imidazole) (**3**) as a slightly yellow solid. The yield was 2.4 g (85%). ^{1}H NMR (500 MHz, DMSO-d_6) δ ppm 4.47 (s, 8H, –CH_2–), 7.10–8.10 (m, 56H, Ar*H*), 12.53 (m, 4H, –N*H*–).

Synthesis of 4,5-diphenyl-2-(4-(prop-2-yn-1-yloxy)phenyl)-1H-imidazole (3).

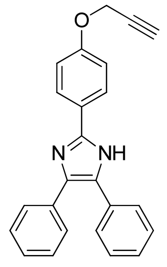

3

Into a flask, benzil (26.3 g, 0.125 mol), 4-(2-propynyloxy)benzaldehyde (20.0 g, 0.125 mmol), ammonium acetate (28.9 g, 0.375 mol), and MeOH (200 mL) were added and the mixture was refluxed for 14 h. After cooling to room temperature, a solid crystallized was collected by filtration washing with cold MeOH. The filtrate was concentrated and the crystallization procedure was repeated twice. The solid obtained was combined and dried under reduced pressure to afford 4,5-diphenyl-2-(4-(prop-2-yn-1-yloxy)phenyl)-1H-imidazole (**3**) as a white solid. The yield was 38.4 g (87%). ^{1}H NMR (500 MHz, $CDCl_3$) δ ppm 2.53 (s, 1H, –C≡C*H*), 4.69 (s, 2H, –OCH_2–), 6.97 (d, 2H, Ar*H*), 7.22–7.33 (m, 6H, Ar*H*), 7.49 (d, 4H, Ar*H*), 7.83 (d, 2H, Ar*H*).

Synthesis of star-PBA$_{Br}$.

Into a flask equipped with three-way cock were placed CuBr (390 mg, 2.73 mmol), dNbpy (2.2 g, 5.6 mmol), and **1** (5.0 g 6.82 mmol), BA (12.3 mL, 137 mmol), and toluene (12

mL), and the mixture was degassed by three “freeze-pump-thaw” cycles. The test tube was placed in an oil bath to proceed the reaction with stirring at 80 °C for 13 h under vacuum. The reaction was then quenched by cooling with liquid nitrogen. After allowed to warm to room temperature, acetone was added to the mixture and the resulting solution was subjected to an alumina column chromatography. The eluent was concentrated and dried under reduced pressure to give slightly yellow oily crude product. A four-armed star PBA (**star-PBA$_{\mathbf{Br}}$**) was obtained by precipitating a chloroform solution of the crude product into DMF. The yield was 10.7 g. ^{1}H NMR (500 MHz, $CDCl_3$) δ ppm 0.94 (s, –CH_2CH_3), 1.16 (s, –C(CH_3)$_2$–), 1.25–2.70 (m, –CH_2CH(COO^{n}Bu)–, –$CH_2CH_2CH_3$), 4.14 (m, -COOCH_2-). M_n (RI) = 1400, M_p (RI) = 1800, M_w/M_n = 1.25. Likewise, **star-PBA$_{\mathbf{Br}}$**s with different molecular weights were synthesized.

Synthesis of star-PBA$_{\mathbf{Azide}}$.

Into a flask, **star-PBA$_{\mathbf{Br}}$** (5.4 g, 2.35 mmol), NaN_3 (2.3 g, 35.4 mmol), and DMF (15 mL) were added and the resulting mixture was stirred at room temperature for 14 h. The reaction mixture was diluted with $CHCl_3$ and poured into an excess amount of water. The aqueous layer was separated and extracted with $CHCl_3$. The combined $CHCl_3$ phases were washed with water, dried over $MgSO_4$, and concentrated to dryness to afford **star-PBA$_{\mathbf{Azide}}$**. The yield was 4.8 g (97%). ^{1}H NMR (500 MHz, $CDCl_3$) δ ppm 0.94 (s, –

CH_2CH_3), 1.16 (s, –C(CH_3)$_2$–), 1.25–2.70 (m, –CH_2CH(COO^{n}Bu)–, –$CH_2CH_2CH_3$), 3.88 (m, –CH_2CH(COO^{n}Bu)N_3), 4.14 (m, -COOCH_2-). M_n (RI) = 1500, M_p (RI) = 1800, M_w/M_n = 1.25.

Synthesis of star-PBA$_{\mathbf{Lophine}}$.

Into a flask, **star-PBA$_{\mathbf{Azide}}$** (3.8 g, 1.5 mmol), **4** (2.2 g, 6.2 mmol), CuBr (890 mg, 6.2 mmol), PMDETA (1.3 mL, 6.2 mmol), and DMF (20 mL) were added and the resulting mixture was stirred at room temperature for 52 h. The reaction mixture was diluted with acetone and passed through a plug of alumina. The eluent was concentrated to dryness and the residue was further purified by silica gel column chromatography using EtOAc/hexane (1/3, v/v) as an eluent to afford **star-PBA$_{\mathbf{Lophine}}$**. The yield was 3.0 g (57%). ^{1}H NMR (500 MHz, $CDCl_3$) δ ppm 0.88 (s, –CH_2CH_3), 1.08 (s, –C(CH_3)$_2$–), 1.21–2.65 (m, –CH_2CH(COO^{n}Bu)–, –$CH_2CH_2CH_3$), 4.05 (m, –COOCH_2–), 5.12 (m, triazole–CH_2OAr), 5.38 (m, –CH_2CH(COO^{n}Bu)–triazole), 6.85–7.93 (–CH_2OArH). M_n (RI) = 2100, M_p (RI) = 2700, M_w/M_n = 1.23.

Synthesis of star-PDMS$_{\mathbf{SiH}}$.

Into a flask was prepared a DMF/THF (30 mL; 2/1, v/v) solution of D3 (3.34 g, 15.0 mmol) and pentaerythritol (255 mg, 7.5 mmol), and a THF solution (1 mL) containing

TBD (10 mg, 0.075 mmol) was added. The mixture was stirred at room temperature for 30 min and toluene solution (40 mL) containing D3 (13.4 g, 60 mmol) was further added. After 22 h, triethylamine (11 mL, 75 mmol) and chlorodimethylsilane (4.17 mL, 37.5 mmol) was added in this order and the mixture was further stirred for 30 min. The mixture was poured into an excess amount of water. The aqueous layer was separated and extracted with hexane/EtOAc (1/1, v/v). The combined hexane/EtOAc phases were washed with water and concentrated. The obtained crude oily product was sequentially washed with DMF and MeOH and dried under reduced pressure to afford **star-PDMS$_{\mathbf{SiH}}$** as a colorless oil. The yield was 11.2 g. ^{1}H NMR (500 MHz, $CDCl_3$) δ ppm 0.03–0.53 (s, –CH_3), 3.59 (s, –CH_2–), 4.72 (m, –SiH). M_n (RI) = 3600, M_p (RI) = 5000, M_w/M_n = 1.78.

Synthesis of star-PDMS$_{\mathbf{Aldehyde}}$.

Into a flask was prepared a toluene solution (20 mL) of **star-PDMS$_{\mathbf{SiH}}$** (5.77 g, 0.54 mmol for end groups) and 4-allyloxy benzaldehyde (1.66 mL, 10.9 mmol), and catalytic amount of xylene solution of Karstedt's catalyst (Pt(dvds)) (50 μL) was added. After stirred at 70 °C for 2.5 h, the mixture was concentrated. The resulting oily product was washed with MeOH and dried under reduced pressure to afford **star-PDMS$_{\mathbf{Aldehyde}}$** as a colorless oil. The yield was 4.85 g. ^{1}H NMR (500 MHz, $CDCl_3$) δ ppm 0.03–0.53 (s, –CH_3), 3.59

(s, –CH_2–), 4.02 (m, –OCH_2–), 7.00 (d, Ar*H*), 7.84 (d, Ar*H*), 9.89 (s, –C*H*O). M_n (RI) = 3600, M_p (RI) = 4800, M_w/M_n = 1.81.

Synthesis of star-PDMS$_{\mathbf{Lophine}}$.

Into a flask containing **star-PDMS$_{\mathbf{Aldehyde}}$** (4.0 g, 1.45 mmol for end groups), benzil (3.05 g, 14.5 mmol), toluene (10 mL), and MeOH (10 mL), ammonium acetate (3.35 g, 43.5 mmol) was added and the resulting mixture was heated at 70 °C for 4 h. After cooling to room temperature, hexane was added and the mixture was poured into an excess amount of DMF. The DMF layer was separated and extracted with hexane. The combined hexane phases were washed with water and MeOH, dried over $MgSO_4$, and evaporation to dryness to afford **star-PDMS$_{\mathbf{Lophine}}$** as a slightly brown oil. The yield was 2.54 g. ^{1}H NMR (500 MHz, $CDCl_3$) δ ppm 0.03–0.53 (s, –CH_3), 0.70 (s, –CH_2CH$_2$CH$_2$OAr), 1.86 (s, –CH$_2$CH_2CH$_2$OAr), 3.59 (m, –CCH_2O–), 3.98 (m, –CH$_2$CH$_2$CH_2OAr), 6.94–8.22 (m, Ar*H*), 9.19 (s, –N*H*–). M_n (RI) = 3700, M_p (RI) = 4800, M_w/M_n = 1.81.

NMR Measurements.

^{1}H NMR spectra were recorded on a Bruker AVANCE III spectrometer operating at 500 MHz. $CDCl_3$, DMSO-d_6 ,or acetone-d_6 was used as the solvent and chemical shifts were reported relative to tetramethylsilane (TMS) (δ = 0.00 ppm) or solvent residual signals.

GPC Measurements.

GPC measurements were performed using a Waters e-2695 high-speed liquid chromatograph equipped with RI and UV detectors. Two series-connected TSKgel SuperMultipore HZ-H columns were employed with tetrahydrofuran (THF) as the eluent at a flow rate of 0.35 mL/min at 40 °C. For polymers containing lophine or HABI groups, THF/TEA (99.5/0.5, v/v) was used as the eluent to avoid interaction between the polymers and column packing materials.

MALDI-TOF Mass Measurement.

MALDI-TOF mass spectra were recorded on a Bruker ultrafleXtreme mass spectrometer equipped with a nitrogen laser (λ = 337 nm). The spectrometer was operated at an accelerating potential of 20 kV with a linear-positive ion mode. THF solutions of a polymer sample (1 mg/mL), dithranol (10 mg/mL) and sodium trifluoroacetate (1 mg/mL) were mixed (1/1/1, v/v/v), and a portion of the mixture was deposited onto a sample target plate.

ESR Measurements.

ESR spectra were recorded on a JEOL JES-TE3000 spectrometer at room temperature. For UV irradiation experiments, UV was directly irradiated to the sample from a window

equipped with sample cavity by using a light guide connected to the Hamamatsu LIGHTNING CURE LC8 L9588.

UV-Vis Absorbance Measurements.

UV-vis absorption spectra were recorded on a JASCO V-560 spectrometer using a quartz cell of 10 mm optical path length. For UV-vis absorbance measurements under the UV irradiation (365 nm, 150 mW/cm^2), the light guide was set inside the sample chamber and UV was directly irradiated to the quartz cells containing THF solutions of **network-PBA$_{HABI}$** (1, 5, 10, 50, and 100 mg/mL).

Supplementary Table 1 | Characterization data for the synthesized **star-PBA$_{\mathbf{Br}}$s**.

Entry[a]	[**1**]/[CuBr]/[dNbpy]/[BA][b]	Conv. (%)[c]	M_n(NMR)[d]	DP_n(arm)[e]	Appearance
1	1/1/1/5	80	2300	3	Liquid
2[f]	1/1/1/5	99	3600	5	Viscous liquid
3	1/1/1/10	98	5500	8	Waxy solid
4	1/1/1/20	99	15900	30	Solid
5	1/1/1/30	98	21500	41	Solid
6	1/1/1/50	99	31700	60	Solid

[a]ATRP of BA conducted with the polymerization time of 2 h. [b]Initial feed ratio. [c]Conversion of BA, determined by ^{1}H NMR. [d]Number averaged molecular weight, determined by ^{1}H NMR. [e]Degree of polymerization per arm, calculated from M_n(NMR). [f]ATRP of BA was conducted with the polymerization time of 5 h.

Supplementary Table 2 | Characterization data for the synthesized **star-PDMS$_{\mathbf{Lophine}}$s**.

Entry	M_n(NMR)^a	M_n(RI)^b	M_w/M_n^{c}	DP$_n$(arm)^d	Appearance
1	20000	3700	1.81	21	Liquid
2	21000	4600	2.29	22	Liquid
3	24700	5400	2.03	26	Liquid

^{a}Number averaged molecular weight, determined by ^{1}H NMR. ^{b}Number averaged molecular weight, determined by GPC with RI detector. ^{c}Molecular weight distribution, determined by GPC with RI detector. ^{d}Degree of polymerization per arm, calculated from M_n(NMR).

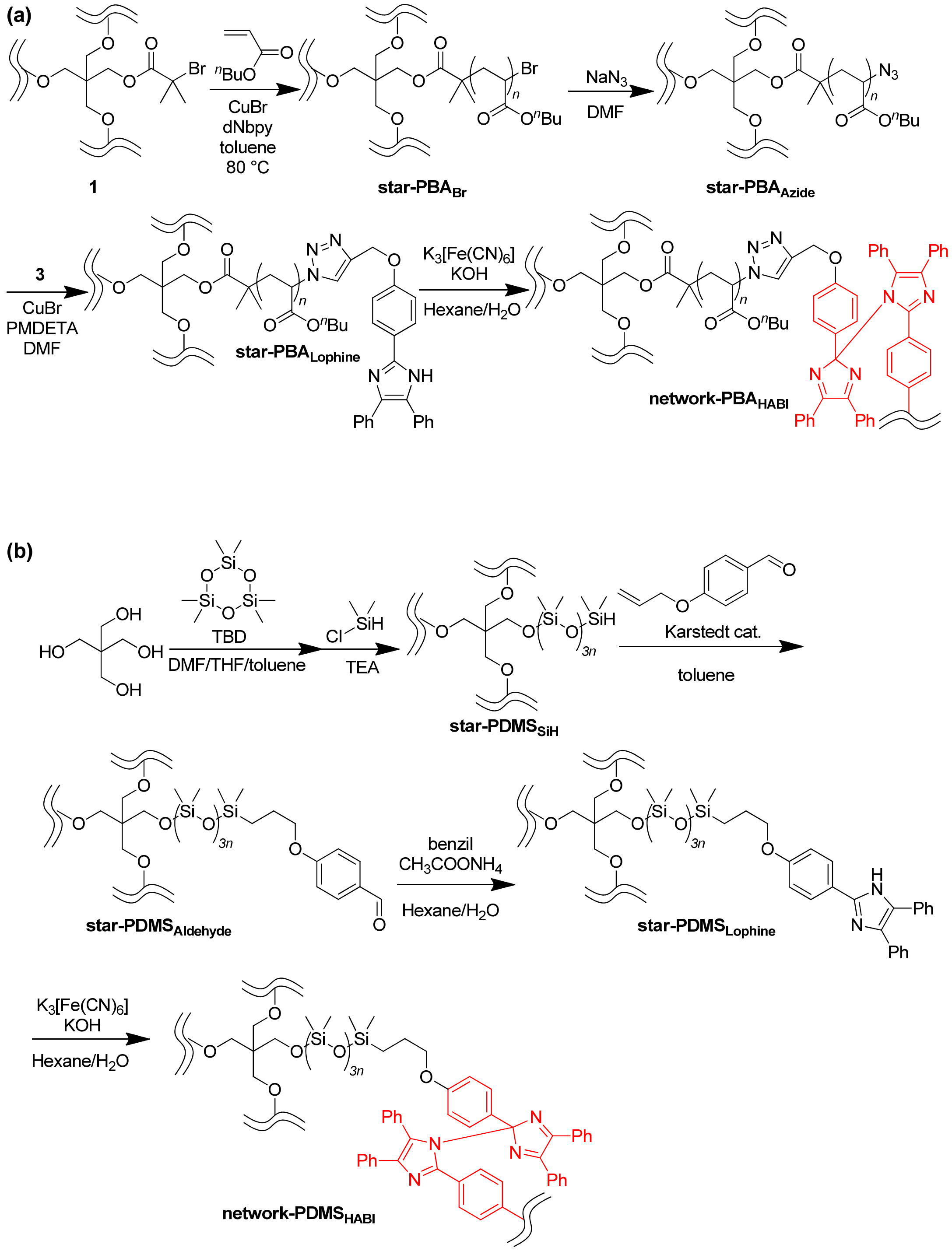

Supplementary Figure 1 | Synthetic routes for (a) **network-PBA$_{\mathbf{HABI}}$** and (b) **network-PDMS$_{\mathbf{HABI}}$**.

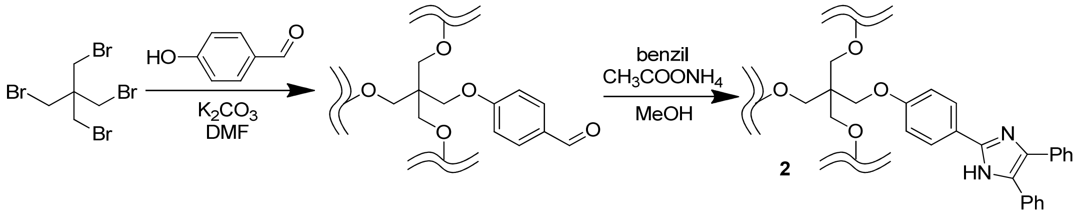

Supplementary Figure 2 | Synthesis of the model small molecule (**2**).

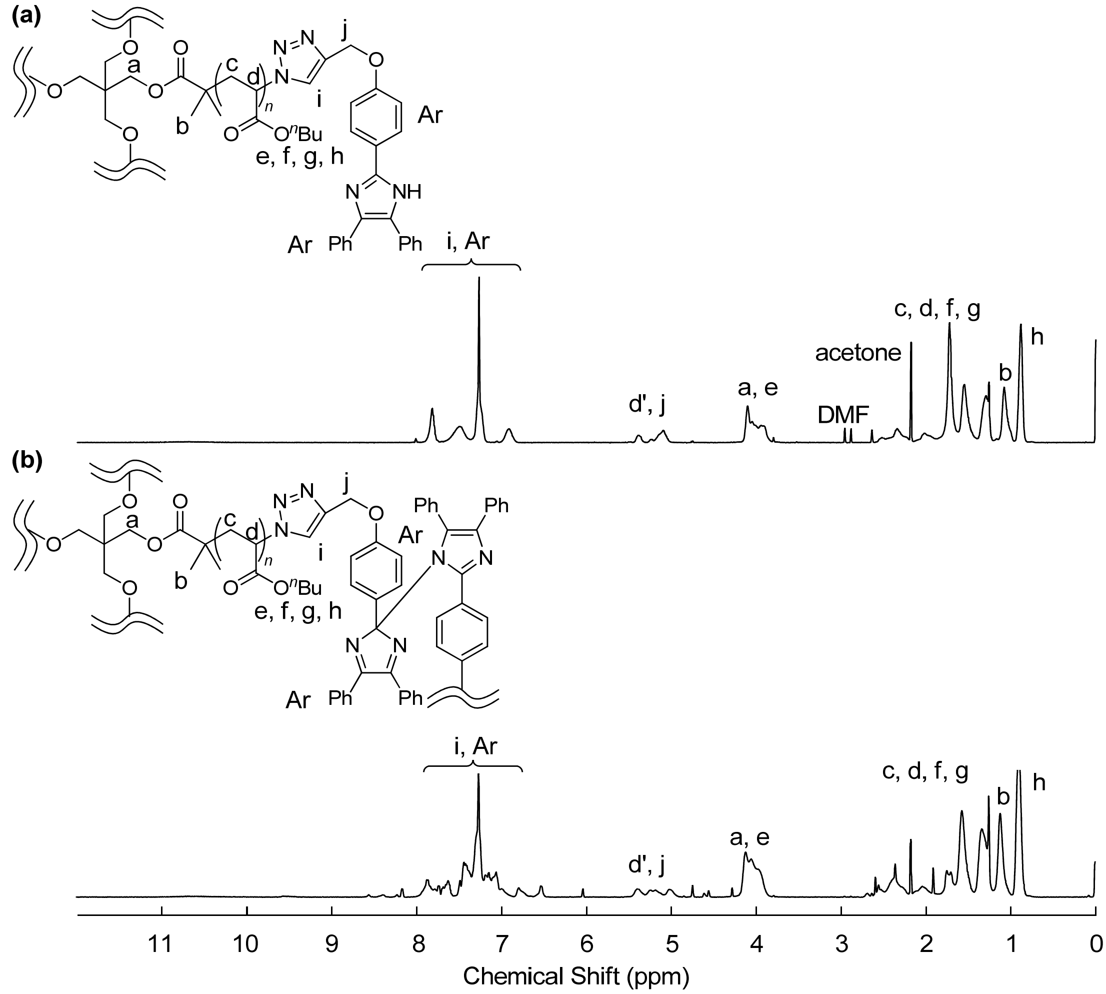

Supplementary Figure 3 | ^{1}H NMR (500 MHz) spectra of (a) **star-PBA$_{\mathbf{Lophine}}$**, and (b) **network-PBA$_{\mathbf{HABI}}$** ($CDCl_3$, 25 °C).

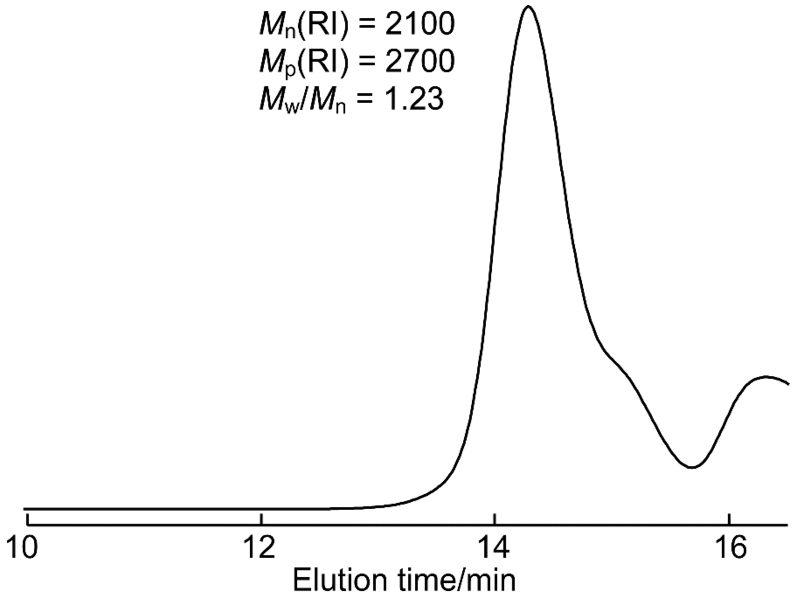

Supplementary Figure 4 | GPC trace of **star-PBA$_{\mathbf{Lophine}}$**.

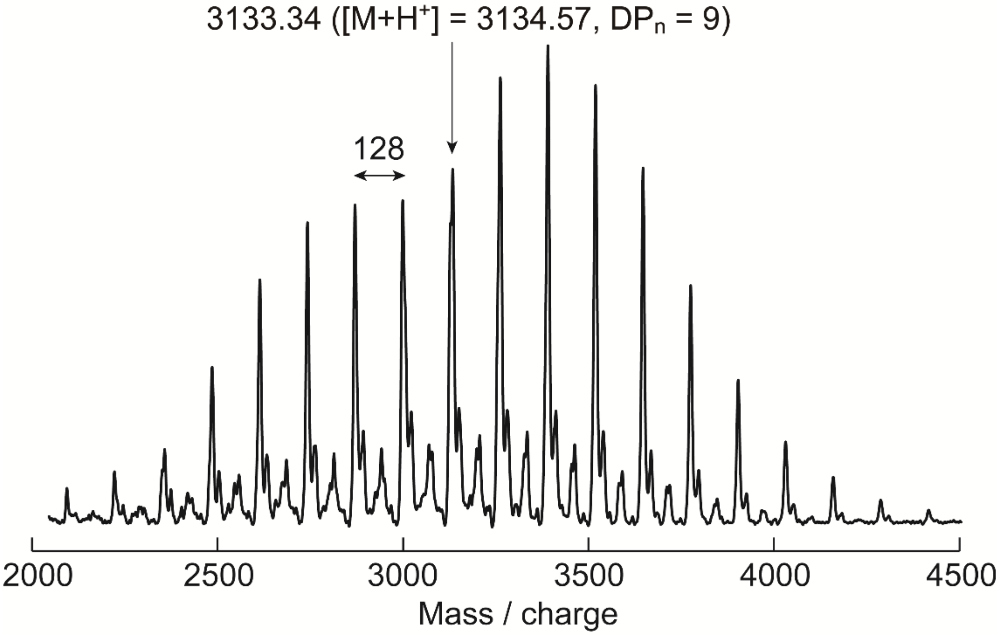

Supplementary Figure 5 | MALDI-TOF mass spectrum of **star-PBA$_{\mathbf{Lophine}}$**

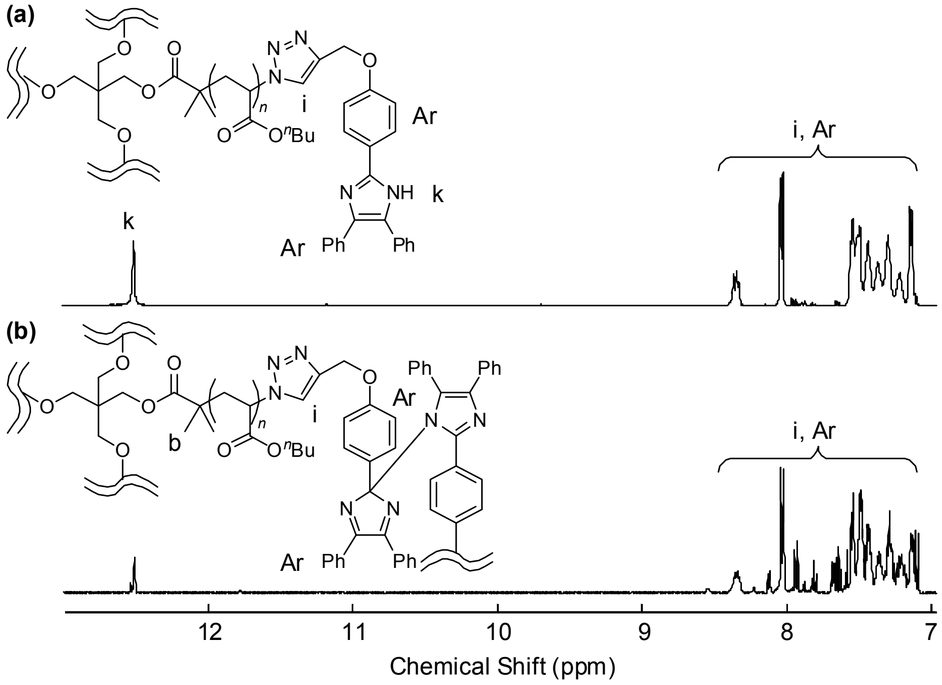

Supplementary Figure 6 | Extended ^{1}H NMR (500 MHz) spectra of (a) **star-PBA$_{Lophine}$** and (b) **network-PBA$_{HABI}$** at aromatic regions (DMSO-d_6, 25 °C).

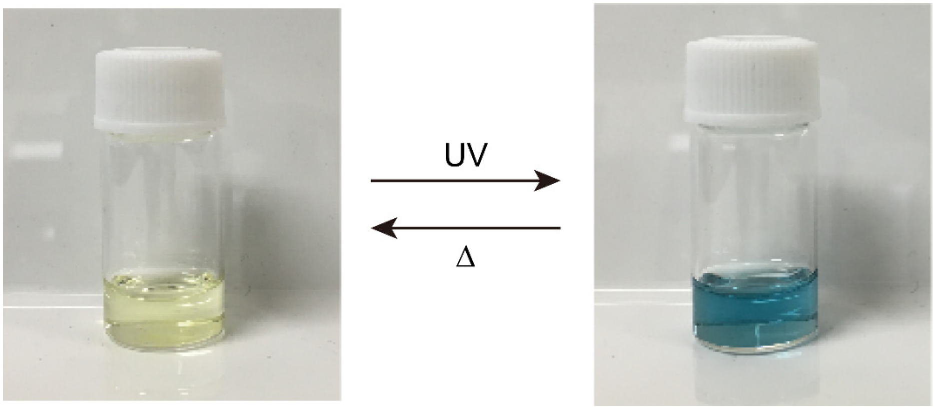

Supplementary Figure 7 | Photographs of THF solutions of (a) **network-PBA$_{HABI}$** and (b) **star-PBA$_{TPIR}$**. The concentration of the solution was 10 mg/mL.

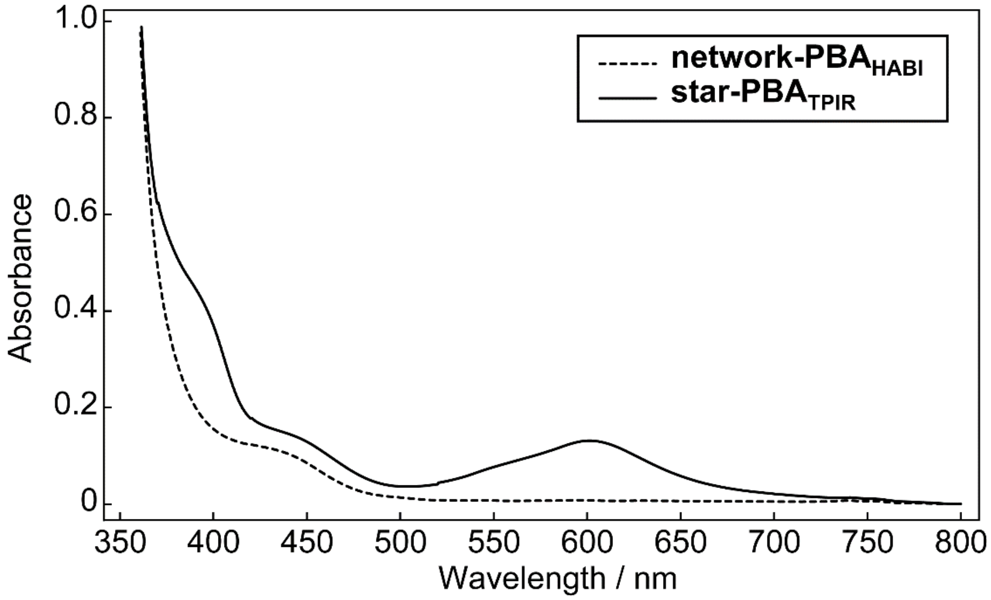

Supplementary Figure 8 | UV-vis spectra of **network-PBA$_{HABI}$** (dashed line) and **star-PBA$_{TPIR}$** (solid line) recorded at the concentration of 5.0 mg/mL.

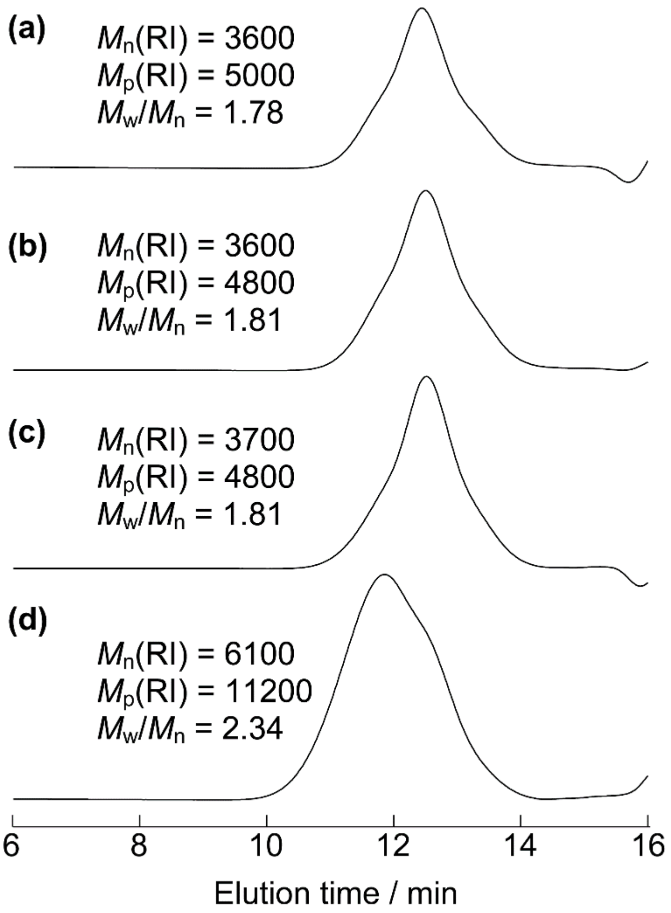

Supplementary Figure 9 | GPC traces of (a) **star-PDMS$_{\mathbf{SiH}}$**, (b) **star-PDMS$_{\mathbf{Aldehyde}}$**, (c) **star-PDMS$_{\mathbf{Lophine}}$**, and (d) **network-PDMS$_{\mathbf{HABI}}$**.

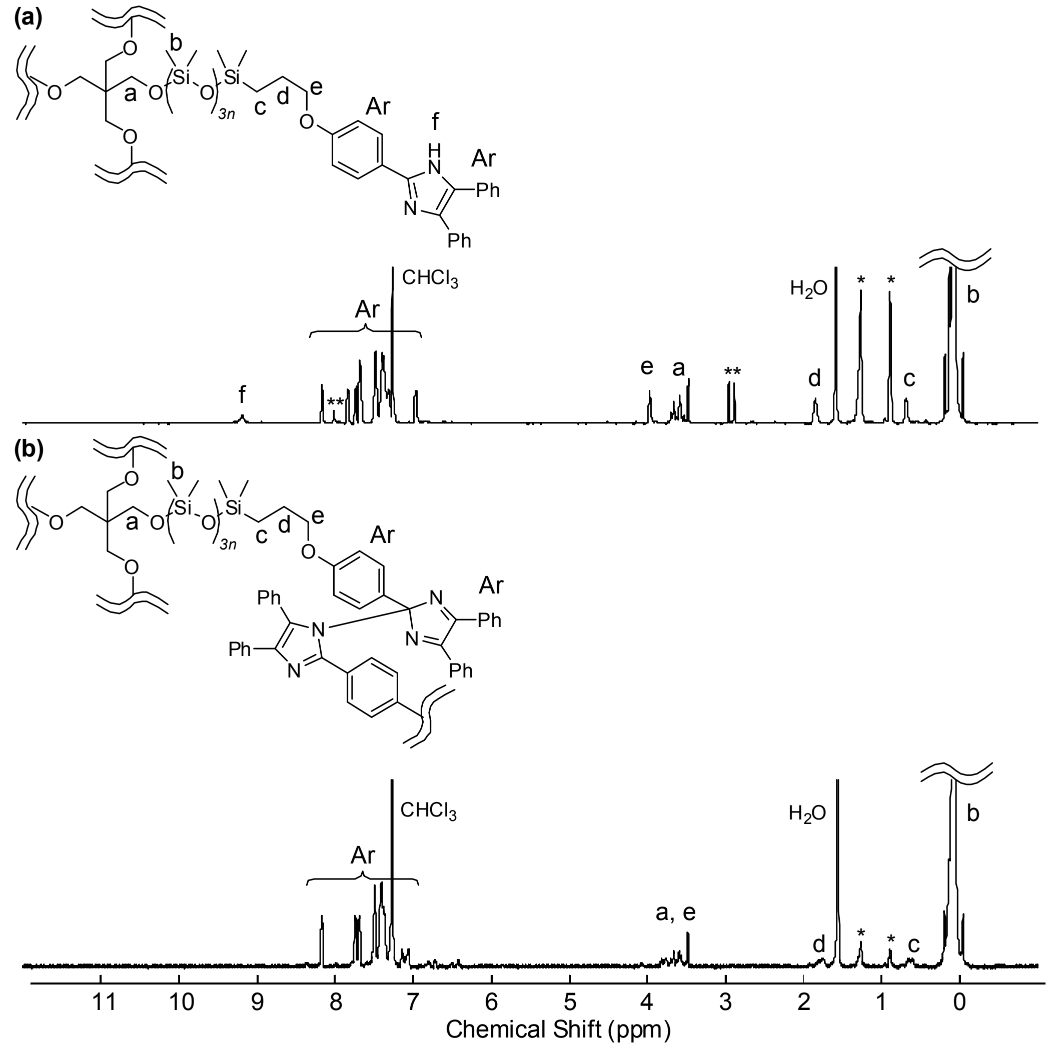

Supplementary Figure 10 | ^{1}H NMR (500 MHz) spectra of (a) **star-PDMS$_{\mathbf{Lophine}}$**, and (b) **network-PDMS$_{\mathbf{HABI}}$**. The signals marked with * and ** are due to the residual hexane and DMF, respectively ($CDCl_3$, 25 °C).

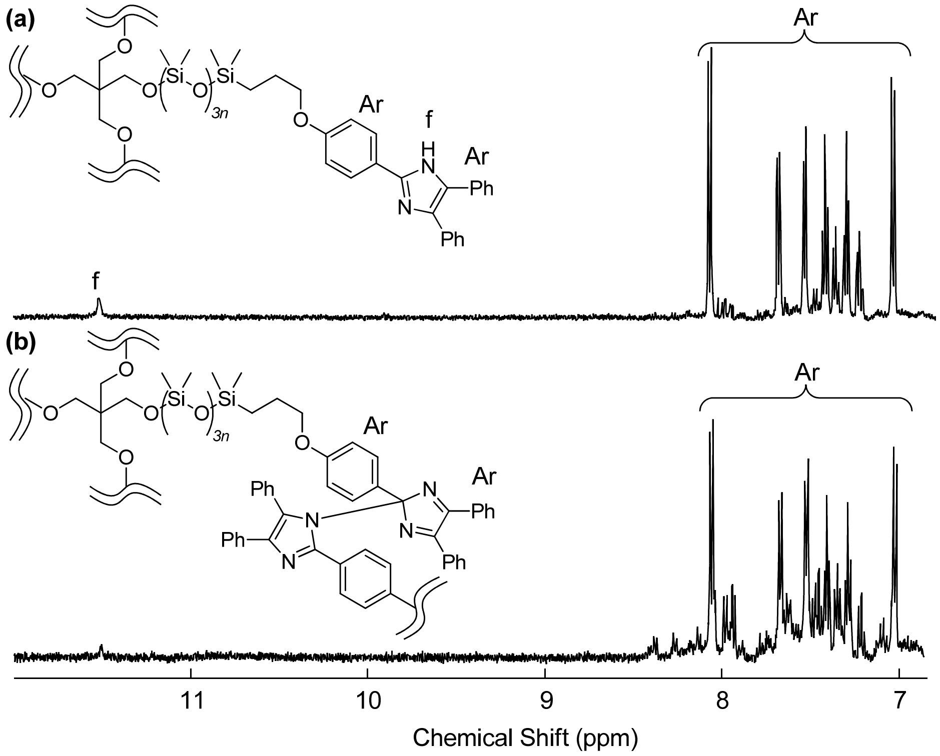

Supplementary Figure 11 | Extended ^{1}H NMR (500 MHz) spectra of (a) **star-PDMS$_{\mathbf{Lophine}}$**, and (b) **network-PDMS$_{\mathbf{HABI}}$** at aromatic regions (acetone-d_6, 25 °C).

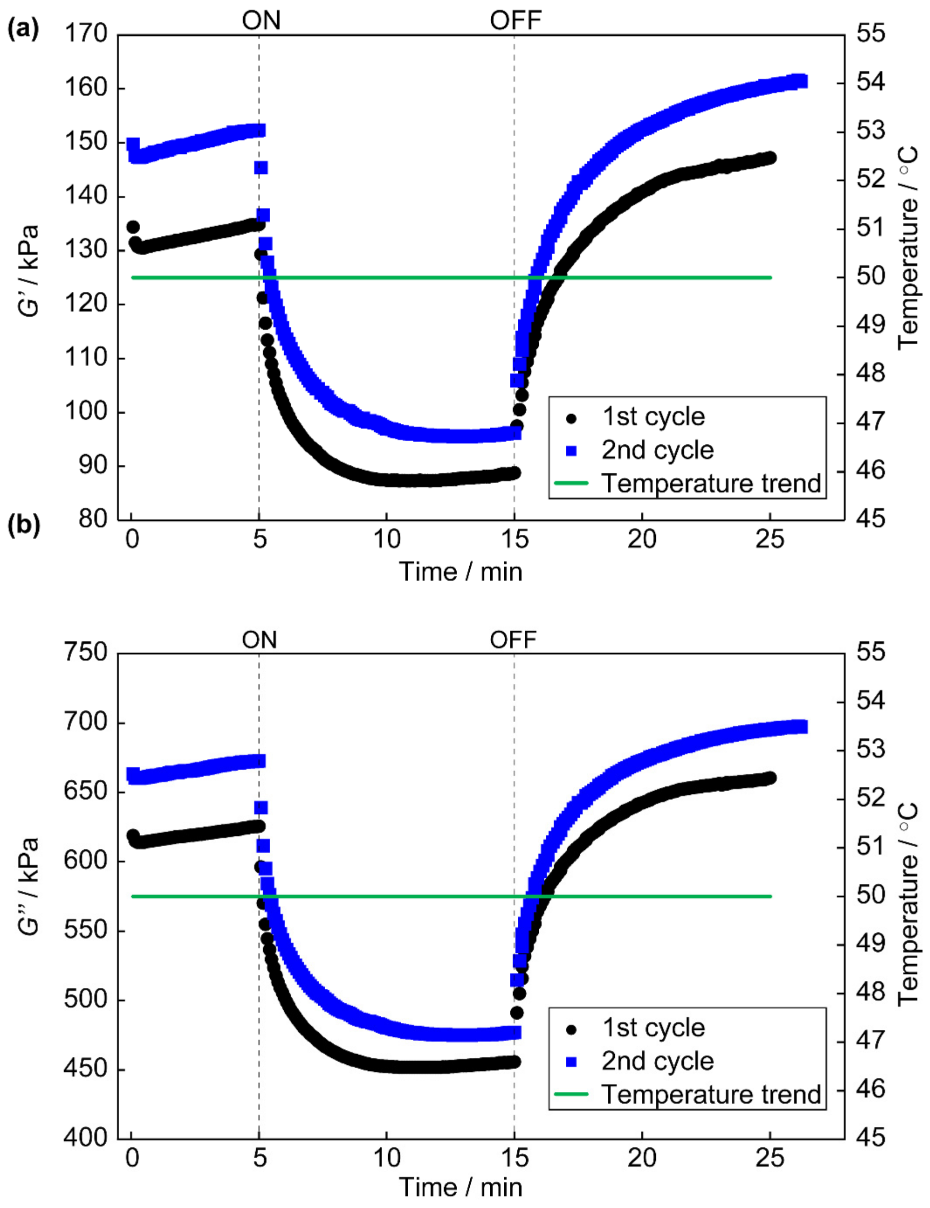

Supplementary Figure 12 | Time-dependent plots of (a) *G'* and (b) *G''* for **network-PBA$_{HABI}$** upon repeating UV irradiation cycles (1st cycle: black circle and 2nd cycle: blue square) with monitoring temperature (green line). The starting (ON) and terminating (OFF) times of UV irradiation were indicated with dashed lines.

Supplementary References

1. Eric, O.-T. et al. Synthesis of AB block and A_2B_2 and A_3B_3 miktoarm star-shaped copolymers using *w*-end-functionalized poly(methyl methacrylate) with a hydroxyl group prepared by organocatalyzed group transfer polymerization. *Polym. Chem.* **6**, 7841–7850 (2015).
2. Gaspar, B. & Carreira, E.M. Mild cobalt-catalyzed hydrocyanation of olefins with tosyl cyanide. *Angew. Chem. Int. Ed.* **46**, 4519–4522 (2007).
